# Vasopressin V2 receptor, tolvaptan, and ERK1/2 phosphorylation in the renal collecting duct

**DOI:** 10.1152/ajprenal.00124.2023

**Published:** 2023-11-02

**Authors:** Shaza Khan, Viswanathan Raghuram, Lihe Chen, Chung-Lin Chou, Chin-Rang Yang, Syed J. Khundmiri, Mark A. Knepper

**Affiliations:** ^1^Epithelial Systems Biology Laboratory, Systems Biology Center, National Heart, Lung, and Blood Institute, National Institutes of Health, Bethesda, Maryland, United States; ^2^Department of Physiology and Biophysics, College of Medicine, Howard University, Washington, District of Columbia, United States

**Keywords:** aquaporin-2, collecting duct, kidney, RNA-seq, tolvaptan

## Abstract

Tolvaptan, a vasopressin antagonist selective for the V2-subtype vasopressin receptor (V2R), is widely used in the treatment of hyponatremia and autosomal-dominant polycystic kidney disease (ADPKD). Its effects on signaling in collecting duct cells have not been fully characterized. Here, we perform RNA-seq in a collecting duct cell line (mpkCCD). The data show that tolvaptan inhibits the expression of mRNAs that were previously shown to be increased in response to vasopressin including aquaporin-2, but also reveals mRNA changes that were not readily predictable and suggest off-target actions of tolvaptan. One such action is activation of the MAPK kinase (ERK1/ERK2) pathway. Prior studies have shown that ERK1/ERK2 activation is essential in the regulation of a variety of cellular and physiological processes and can be associated with cell proliferation. In immunoblotting experiments, we demonstrated that ERK1/ERK2 phosphorylation in mpkCCD cells was significantly reduced by vasopressin, in contrast to the increases seen in non-collecting-duct cells overexpressing V2R in prior studies. We also found that tolvaptan has a strong effect to increase ERK1/ERK2 phosphorylation in the presence of vasopressin and that tolvaptan’s effect to increase ERK1/ERK2 phosphorylation is absent in mpkCCD cells in which both protein kinase A (PKA)-catalytic subunits have been deleted. Thus, it appears that the tolvaptan effect to increase ERK activation is PKA-dependent and is not due to an off-target effect of tolvaptan. We conclude that in cells expressing V2R at endogenous levels: *1*) vasopressin decreases ERK1/ERK2 activation; *2*) in the presence of vasopressin, tolvaptan increases ERK1/ERK2 activation; and *3*) these effects are PKA-dependent.

**NEW & NOTEWORTHY** Vasopressin is a key hormone that regulates the function of the collecting duct of the kidney. ERK1 and ERK2 are enzymes that play key roles in physiological regulation in all cells. The authors used collecting duct cell cultures to investigate the effects of vasopressin and the vasopressin receptor antagonist tolvaptan on ERK1 and ERK2 phosphorylation and activation.

## Graphical abstract

**Figure:**
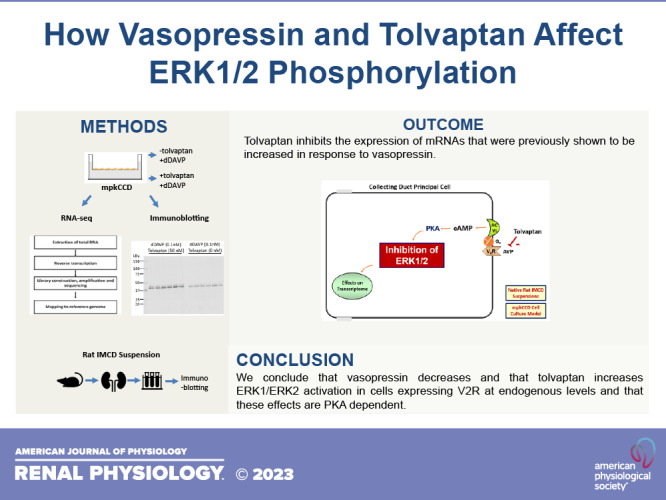

## INTRODUCTION

Arginine vasopressin (AVP), also known as antidiuretic hormone (ADH), is a 9-amino acid peptide hormone that increases water reabsorption in the kidney. It does so mainly by controlling the osmotic water permeability of collecting duct cells via regulation of the water channel aquaporin-2 (AQP2) (1). The action of AVP occurs via its binding to the V2 subtype vasopressin receptor (V2R), a G protein-coupled receptor (GPCR) located on the basolateral plasma membrane of principal cells of the renal collecting ducts. It promotes the reabsorption of water by stimulating adenylyl cyclase 6 (2) via the stimulatory G protein (G_α_s), thereby increasing the production of the second messenger cyclic AMP (cAMP) (3–5). This activates protein kinase A (PKA), which phosphorylates several proteins including AQP2 (6–9) regulating the processes that control the osmotic water permeability. In addition, vasopressin causes intracellular Ca^2+^ mobilization in the form of aperiodic Ca^2+^ spikes (10) through V2R-G_α_s-Adcy6-cAMP-PKA signaling and phosphorylation of the inositol trisphosphate receptor (7). Prior studies in native inner medullary collecting ducts showed that vasopressin-stimulated Ca^2+^ mobilization in inner medullary collecting ducts (IMCD) occurred in the absence of activation of the phosphoinositide pathway (11), which argues against the coupling of Gq/11 with V2R in native collecting ducts. V2R-G_α_s-Adcy6-cAMP-PKA signaling has also been seen to decrease ERK1 and ERK2 activation in native collecting ducts, which is thought to occur in part through PKA-dependent phosphorylation and inhibition of Raf1 (10, 12). In contrast, signaling from the V2 receptor overexpressed in HEK293 cells has been seen to increase, not decrease, ERK1 and ERK2 phosphorylation (13). In another cell line expressing V2R, LLC-PK1 stably expressing c-myc-tagged AQP2, treatment with AVP also seemed to increase ERK phosphorylation (14).

Activation of ERK1 and ERK2 is essential in regulating a wide variety of cellular and physiological processes, including cell differentiation, metabolism, proliferation, and development in many tissues (15). ERK activity can also stimulate entry into the cell cycle and promote or inhibit cell survival, depending on the cellular context and the degree of ERK activation (16, 17). In addition, ERK plays roles in cell movement, cytoskeletal regulation, gene expression, and regulation of transcription (17).

Like other GPCRs, V2R can also activate β-arrestin, which results in ERK1 and ERK2 activation independently of G protein signaling (13, 18). For this to occur, the V2 receptor must be phosphorylated in its COOH-tail by one or more G protein-coupled receptor kinases (GRKs), allowing the receptor to bind β-arrestin (13). Based on phosphoproteomic studies in rat inner medullary collecting ducts (19) and V2R-transfected HEK 293 cells (20), it is known that the V2 receptor can be phosphorylated at several sites in its COOH-terminal tail, although phosphorylation at these sites was not found to increase with vasopressin exposure (19). In addition to ERK activation, β-arrestin signaling results in receptor desensitization and internalization (21). Theoretically, under some circumstances, activation of β-arrestin may dominate over the PKA-mediated inhibition of ERK (18). Activation of ERK can lead to phosphorylation of ETS and AP-1 family transcription factors and result in a proliferative response (22–24).

Water balance disorders associated with V2R-mediated hyperstimulation of collecting duct water permeability and systemic water retention result in dilutional hyponatremia. Hyponatremia is common in hospitalized patients (25, 26). Treatment of hyponatremia has benefitted from the development of V2R antagonists such as tolvaptan (27). The vaptan family of V2R antagonists, like satavaptan and tolvaptan, function as inverse agonists (28, 29). An inverse agonist is an antagonist that can block GPCR ligand-induced activation but can also have ligand-independent effects by suppressing the constitutive activity of the receptor. In prior studies in cells overexpressing V2R (28), it was shown that satavaptan, acting in the absence of vasopressin, can recruit β-arrestin and stimulate ERK1 and ERK2. Thus, vaptans have the potential to promote cell proliferation via ERK activation.

The response of collecting duct principal cells to vasopressin consists of two major elements: *1*) Increased net trafficking of AQP2 to the plasma membrane (30), consisting of increased exocytosis of AQP2-containing vesicles (31, 32) and decreased endocytosis of AQP2 (31–34); and *2*) Increased AQP2 abundance due to transcriptional regulation (35) and increased AQP2 protein stability (36, 37). A cell line derived from mouse collecting duct principal cells (mpkCCD) has been shown to replicate both elements of the collecting duct vasopressin response and to reproduce the pattern of AQP2 phosphorylation seen in native collecting duct cells (38). This establishes that these cells have endogenous V2 receptor activity. Since many prior studies characterizing V2 receptor signaling have been carried out in non-collecting duct cell lines (chiefly HEK293 cells) overexpressing the V2 receptor and other signaling components, conclusions from such studies may not be directly extrapolatable to collecting duct cells. Here we use RNA-seq in the mpkCCD cell line to ask whether tolvaptan alters the transcriptome of mpkCCD cells in a manner predictable from the known actions of vasopressin working through the V2 receptor, or whether there are unexpected effects that would point to off-target or non-canonical actions? Given evidence from the RNA-seq results that point to a strong perturbation of the ERK signaling pathway, we ask the following additional questions: *1*) What are the effects of AVP and dDAVP on ERK1 and ERK2 phosphorylation/activation? *2*) What is the effect of tolvaptan on ERK1 and ERK2 phosphorylation/activation in the presence and absence of dDAVP? and *3*) What is the role of PKA in vasopressin-mediated regulation of ERK1 and ERK2 phosphorylation/activation and on the effect of tolvaptan?

## METHODS

### Cell Culture of the mpkCCD Clonal Cell Line

The original mpkCCD line was derived from micro-dissected cortical collecting ducts from SV-PK/Tag transgenic mice (39). These cells were re-cloned to obtain a subclone (clone 11) that expresses AQP2 at a high level and displays regulation of trafficking and AQP2 abundance by AVP (38). Experiments were performed in mpkCCD11 cells (passages 8–12). In some experiments, mpkCCD cells were also used in which both *Prkaca* and *Prkacb* gene expression was deleted using CRISPR-Cas9 to generate PKA double knockout (PKA dKO) cell lines. Control cell lines, referred to as “PKA intact” underwent the CRISPR-Cas9 protocol but did not exhibit the deletion of the PKA catalytic gene (7). Cells were expanded to ∼80% confluence on 25-cm^2^ plastic flasks (Corning Costar), trypsinized (0.05% trypsin, 1.5 mM EDTA), and resuspended in 10 mL DMEM/F12. Then cells were seeded on permeable supports (24-mm^2^ diameter, 0.4-μm pore size, Corning Costar, Cat. No. 3450) at a ratio of 1:10 and grown in 1:1 DMEM/F12 (Invitrogen) containing 2% heat-inactivated fetal bovine serum (catalog no. 100–106, BenchMark, Gemini Bio-Products) and the following supplements: 5 μg/mL insulin, 50 nM dexamethasone, 1 nM triiodothyronine, 5 μg/mL transferrin, 10 ng/mL epidermal growth factor (EGF), and 60 nM sodium selenite, all from Sigma. All cells were cultured at 37°C and 5% CO_2_ in a humidified incubator. The vasopressin V2 receptor-selective agonist 1-desamino-8-d-arginine-vasopressin (dDAVP, 0.1 nM) was added to the basolateral medium to stimulate the expression of AQP2 and other vasopressin-responsive proteins. Apical and basolateral media were changed daily. A day before the experiment, the cells were put into a minimal medium DMEM/F12 (Invitrogen) deprived of serum, hormones, and growth factors containing supplements (50 nM dexamethasone, 5 μg/mL transferrin, and 60 nM sodium selenite) to ensure complete polarization. On the day of the treatment and cell harvest, the basolateral medium was replaced by a fresh minimal medium containing the treatment of choice; tolvaptan, dDAVP, tolvaptan plus dDAVP simultaneously, AVP, EGF, or vehicle for 30 min. Tolvaptan (molecular mass of 448.94 g/mol) was dissolved in DMSO and glycerol and stored as 1,000× stock solutions in a −20°C freezer until used. Samples were lysed with Laemmli buffer (1.5% SDS, 10 mM Tris, pH 6.8, 1× Halt protease and phosphatase inhibitor cocktail, EDTA-free [Thermo Scientific, NY]) and collected and passed through QIAshredder (Qiagen). Protein concentration was measured using the BCA Protein Assay Kit (Thermo Fisher Scientific). Samples were added with 5× loading buffer [7.5% (wt/vol) SDS, 30% (vol/vol) glycerol, 200 mM DTT, 50 mM Tris, bromophenol blue, pH 6.8] and incubated at 65°C for 10 min for immunoblotting.

### Immunoblot Analysis

The denatured protein samples (10 μg) were subjected to SDS/PAGE gel electrophoresis on 4–12% polyacrylamide gels (BioRad) and transferred electrophoretically onto nitrocellulose membranes. The membranes were then blocked with Odyssey blocking buffer (LI-COR), and probed overnight at room temperature with primary antibodies; phospho-p44/42 MAPK (ERK1/2) (Thr^202^/Tyr^204^) or total p44/42 MAPK (ERK1/2) (Cell Signaling Technology), used at 1:1,500–2,000 dilution. Separate immunoblots were done for total and phospho-ERK. After washing, blots were incubated with infrared fluorescence-conjugated secondary antibodies used at 1:5,000 for the detection of all primary antibodies. Both blocking buffer and IR dye-coupled secondary antibodies were obtained from LI-COR (Lincoln, NE). Fluorescence was imaged and quantified using a LI-COR Odyssey Imaging System.

### Densitometry and Statistical Analysis

A net intensity value of each immunoblot band was quantified using the LI-COR Image Studio software. Both the GraphPad Prism software and the “data analysis” add-in within Microsoft Excel were utilized for analyzing the data. Each experimental value had its own contemporaneous control. For non-time course experiments, as appropriate, unpaired *t* test or one-way ANOVA with Tukey contrasts was performed. *P* value <0.05 was considered statistically significant.

### RNA Sequencing and Data Analysis

Cells on the 6-well inserts were washed twice with cold PBS before adding 1 mL TRizol for RNA extraction. 60 µL of the TRizol lysate was used for total RNA purification. RNA was eluted in 50 µL water and 1 µL of the eluted RNA was used for cDNA generation using the SMARTer V4 Ultra Low RNA Kit (Takara Bio) (40, 41). cDNA was generated with 8 PCR cycles. 1 ng of the resulting cDNA was “tagmented” and barcoded by using the Nextera XT DNA Sample Preparation Kit (Illumina) to generate sequencing libraries for high throughput sequencing. Libraries were purified by AmPure XP magnetic beads (Beckman Coulter) and quantified using a Qubit 2.0 Fluorometer (Thermo Fisher Scientific). An equal amount of index libraries were pooled and sequenced (paired-end 50 bp) on an Illumina NovaSeq 6000 platform.

RNA-seq raw sequencing reads were aligned by STAR (42) to the mouse Ensemble genome (release 106). Transcript per million (TPM) and expected counts were generated by RSEM (43). All samples are of a high percentage of uniquely mapped reads (>80%) and are included for downstream analysis. Unless otherwise specified, the computational analyses were performed on the National Institutes of Health Biowulf High-Performance Computing platform.

Differential gene expression (DGE) analysis was performed using the R package DEseq2 (44). A pre-filtering was done to ensure at least 4 samples with a count of 10 or more. Were kept as differentially expressed genes (DEGs, *n* = 23). Gene Ontology (GO) was done in DAVID (Database for Annotation, Visualization and Integrated Discovery, NIAID, Bethesda, MD) Metascape (45).

### Data Availability

Sequencing data including the raw processing file have been deposited in the Gene Expression Omnibus (GEO, GSE243773).

### IMCD Suspensions

Male Sprague-Dawley rats were obtained from Taconic Bioscience and had free access to water and a rodent diet before the experiment. The rats were injected intraperitoneally with furosemide (5 mg/rat) 20 min before euthanasia by decapitation. Furosemide is used to reduce osmotic shock to renal tissue by dissipating the hypertonic medullary osmolality (46). The inner medullas were isolated as described (47), minced, and digested in an enzyme solution (3 mg/mL collagenase B, 2,000 U/mL hyaluronidase, 250 mM sucrose, 10 mM triethanolamine, pH 7.6) with continuous stirring for 90 min at 37°C. The resulting suspension was sedimented by low-speed centrifugation at 70 *g* for 20 s, separating them from the lighter non-IMCD fragments. The supernatant was discarded, and the pellet was resuspended in tubule suspension fluid containing 118 mM NaCl, 25 mM NaHCO_3_, 5.5 mM glucose, 5 mM KCl, 4 mM Na_2_HPO_4_, 2 mM CaCl_2_, and 1.2 mM MgSO_4_, pH 7.4, equilibrated with 5% CO_2_/95% air. This low-speed centrifugation and wash procedure was repeated twice. After removal of the supernatant, the IMCD fragments were resuspended with the suspension fluid, divided into 8 aliquots in polypropylene tubes (4 control and 4 experimental), and warmed to 37°C before studies. The suspensions were treated with tolvaptan (50 nM) (*n* = 4) or vehicle (*n* = 4) for 30 min, then dDAVP (0.1 nM) for 2 min for both groups. The reaction was arrested and followed by centrifugation at 2000 *g* for 1 min. The pellets were homogenized in Laemmli buffer (1.5% SDS, 10 mM Tris, pH 6.8, protease and phosphatase inhibitors) and passed through QIAshredder (Qiagen). Protein concentration was measured using BCA Protein Assay Kit (Thermo Fisher Scientific). Immunoblotting was performed as described previously.

## RESULTS

### Effect of Tolvaptan on the mpkCCD Transcriptome

**Figure 1.**
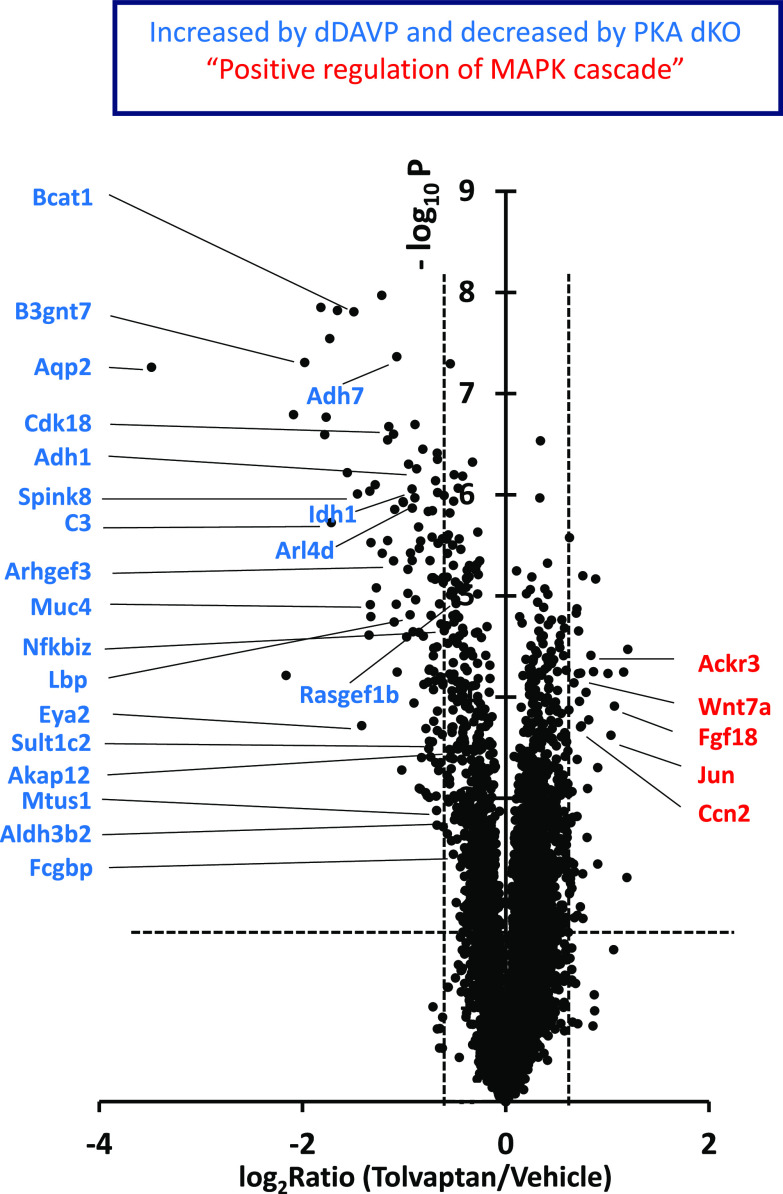
Volcano plot representation of transcriptomic changes in mpkCCD collecting duct cells after 24-h exposure to tolvaptan. *Blue,* increased by dDAVP and decreased by PKA dKO; *Red*, Gene Ontology Biological Process term “positive regulation of MAPK cascade.” PKA dKO, protein kinase A double knockout.

**Figure 2.**
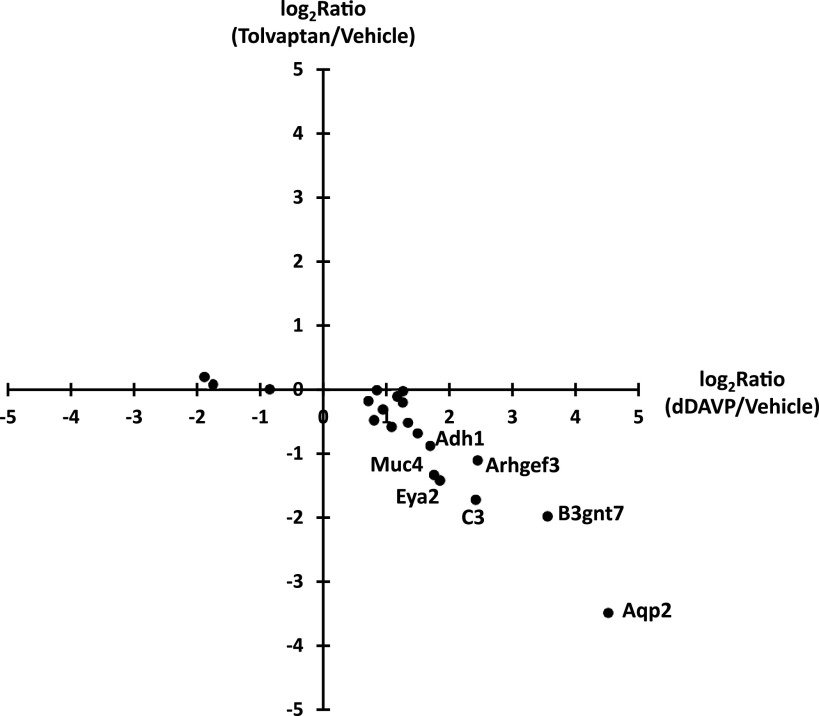
Plot showing log_2_ (dDAVP/vehicle) values for RNA-seq in mpkCCD from prior study (35) vs. log_2_ (tolvaptan/vehicle) values in the current study.

**Figure 3.**
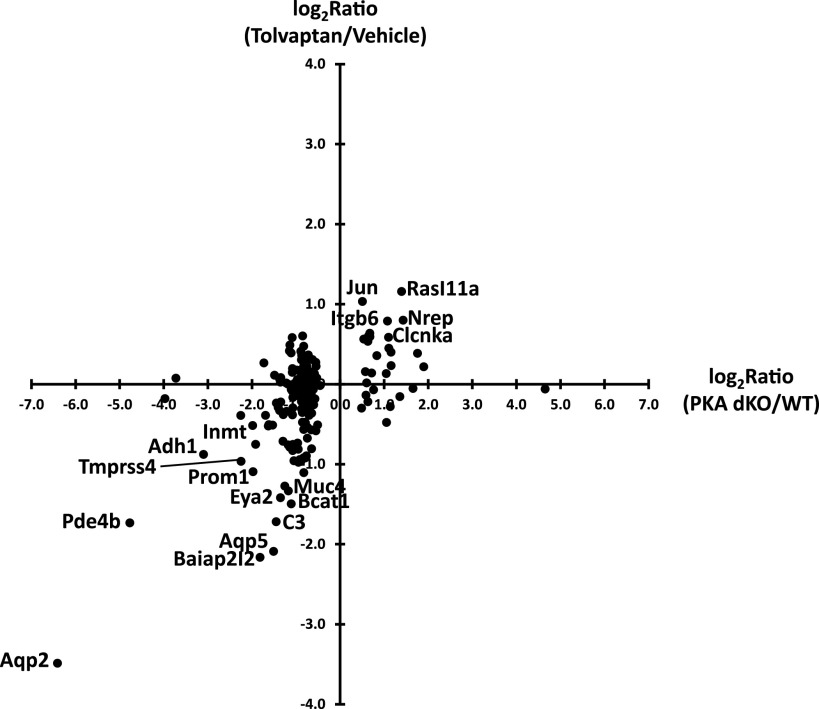
Plot showing log_2_ (PKA dKO/vehicle) values for RNA-seq from prior study (7) in double knockout (dKO) cells (both PKA catalytic genes deleted using CRISPR-Cas9) generated from mpkCCD cell line vs. log_2_ (tolvaptan/vehicle) values in the current study. PKA dKO, protein kinase A double knockout.

To assess the effect of tolvaptan on the transcriptome of cultured collecting duct cells (mpkCCD), we used RNA-seq. The cells were grown to confluence on permeable supports in the presence of dDAVP (0.1 nM). With the continued presence of dDAVP, cells were exposed to tolvaptan (50 nM) or its vehicle for 24 h and harvested for RNA-seq analysis. The filtered data (Maximum TPM > 10, *n* = 9,678 transcripts) are provided as a publicly accessible web page at https://esbl.nhlbi.nih.gov/Databases/Tolvaptan-transcriptome/. Using stringent criteria (|Log_2_(tolvaptan/control)| > 0.593 and *P* < 0.01), 112 transcripts were identified as decreased and 51 were identified as increased. (The range [−0.593, 0.593] defines the 95% confidence interval based on the distribution of all (|Log_2_(tolvaptan/control)| values [empirical Bayes method]). The overall false discovery rate (FDR) is therefore 0.01 × 0.05 = 0.0005. Figure 1 shows a volcano plot representation of the results. The data are skewed somewhat toward decreased TPM levels, with many of the decreased transcripts having been previously identified as being increased in response to dDAVP in prior studies (35) and decreased in PKA dKO cells (7). Also indicated in Fig. 1 are transcripts matching the Gene Ontology Biological Process term “positive regulation of MAPK cascade.” Figure 2 plots the prior dDAVP responses (35) versus the tolvaptan responses in the current study showing a high degree of concordance. Figure 3 plots the prior responses to PKA deletion (knockout of both catalytic subunits, dKO) (7) versus the tolvaptan responses, also demonstrating a high degree of concordance. However, there were many additional transcripts that exhibited changes that were not previously seen in response to dDAVP or PKA deletion. To identify processes associated with these additional perturbed transcripts we used DAVID (Database for Annotation, Visualization and Integrated Discovery, NIAID, Bethesda, MD) to ask what Gene Ontology Biological Process (GO-BP) terms displayed over-representation of transcripts that were either decreased (Table 1) or increased (Table 2).

**Table 1.** Gene Ontology Biological Process terms for which transcripts downregulated in response to tolvaptan in mpkCCD cells are over-represented*

GO-BP Term	Fisher Exact *P*	Fold Enrichment	Transcripts
G-protein coupled receptor signaling pathway	0.000003	4.77	BCAR3, RAMP3, CAV2, FZD4, SLC39A14, AKAP12, C3, RGS2, KCNQ1, ADORA1, FFAR4, RAMP1, TGM2
Regulation of inflammatory response	0.00032	3.76	C3, NFKBIZ, ADORA1, FFAR4, LBP, LGALS9, PTGS2, TRIM65, IER3, PTGES
Negative regulation of cell adhesion	0.0027	3.35	ARG2, DMTN, FZD4, LGALS9, CCL28, B4GALNT2, MUC4, TNFRSF21
Anion transport	0.0023	2.74	ABCC3, RGS2, ARG2, SLC35A3, CRABP2, KCNQ1, ADORA1, ACSL4, PTGS2, PTGES, SLC25A13
Negative regulation of transport	0.001	2.70	DMTN, USP2, ACSL4, PTGS2, SMIM6, TBC1D1, RGS2, KCNQ1, ADORA1, FFAR4, CHP1, LGALS9, IER3
Cation transport	0.000022	2.66	ARG2, RAMP3, SLC31A1, USP2, SLC35G1, AQP2, PTGS2, SMIM6, SLC5A3, SLC39A14, RGS2, FXYD4, NIPAL1, KCNQ1, PDE4B, ADORA1, CHP1, ATP6V0A4, SIK1, RAMP1, SLC39A4, TGM2
Response to peptide hormone	0.0091	2.27	NR4A2, BCAR3, GHR, SOCS2, NR4A1, CITED1, GRB14, CAV2, KCNQ1, PTGS2, SLC39A14
Transmembrane receptor protein tyrosine kinase signaling pathway	0.0069	2.26	CYFIP2, BCAR3, GHR, SOCS2, BTC, GRB14, SLC31A1, CAV2, TNK1, ITGA1, ADORA1, SLC39A14
Regulation of MAPK cascade	0.0073	2.15	BCAR3, RAMP3, CAV2, FZD4, ITGA1, AKAP12, GHR, C3, RGS2, ADORA1, FFAR4, STK40, AVPI1
Negative regulation of cell proliferation	0.0099	2.07	ARG2, SLFN2, CAV2, ITGA1, PTGS2, NDRG1, TOB1, DNAJB2, FBXO4, ADORA1, LGALS9, TNFRSF21, PTGES
Signal transduction by protein phosphorylation	0.03	1.84	AKAP12, BCAR3, GHR, C3, RGS2, RAMP3, CAV2, FZD4, ITGA1, ADORA1, FFAR4, AVPI1
Cell surface receptor signaling pathway	0.014	1.50	CYFIP2, BCAR3, CITED1, TOB1, OAS1F, GHR, SOCS2, C3, GRB14, NFKBIZ, PDE4B, ADORA1, LGALS9, LBP, TRIM65, BCL2L14, FYB2, DMTN, FZD4, EYA2, CAV2, SLC31A1, ITGA1, TNK1, SLC39A14, BTC, KRT19, PTP4A3, NCEH1, TNFRSF21

*Calculations used DAVID (NIAID, https://david.ncifcrf.gov/). DAVID, Database for Annotation, Visualization and Integrated Discovery; GO-BP, Gene Ontology Biological Process.

**Table 2.** Gene Ontology Biological Process terms for which transcripts upregulated in response to tolvaptan in mpkCCD cells are over-represented*

GO-BP Term	Fisher Exact *P*	Fold Enrichment	Transcripts
Response to transforming growth factor beta	0.000000073	13.6	TGFBR3L, JUN, RASL11B, SCX, WNT7A, ITGB6, NREP, DBN1
Positive regulation of epithelial cell proliferation	0.00052	10.5	JUN, FGF18, WNT7A, GPBAR1
ERK1 and ERK2 cascade	0.00011	10.2	JUN, FGF18, GPBAR1, ACKR3, CCN2
Positive regulation of MAPK cascade	0.00013	7.4	JUN, FGF18, WNT7A, GPBAR1, ACKR3, CCN2
Kidney development	0.004	6.1	FRAS1, COL4A4, SOX4, FREM2
Positive regulation of cell proliferation	0.0015	4.0	JUN, FGF18, SCX, WNT7A, GPBAR1, CCN2, SOX4
Cell surface receptor signaling pathway	0.000019	3.3	JUN, RASL11B, SCX, WNT7A, GPBAR1, NREP, TGFBR3L, FGF18, ACKR3, NRTN, ITGB6, CCN2, DBN1, SOX4
Regulation of apoptotic process	0.019	2.3	JUN, VNN1, SCX, WNT7A, EPC1, ACKR3, CCN2, SOX4
Animal organ development	0.0017	2.2	JUN, SCX, WNT7A, TGFBR3L, FRAS1, VNN1, COL4A4, FGF18, NRTN, ITGB6, CCN2, MYH10, SOX4, FREM2

*Calculations used DAVID (NIAID, https://david.ncifcrf.gov/). DAVID, Database for Annotation, Visualization and Integrated Discovery; GO-BP, Gene Ontology Biological Process.

Table 1 includes several GO-BP terms that are expected based on known actions of vasopressin, namely “G-protein coupled receptor signaling pathway,” “response to peptide hormone,” “cell surface receptor signaling pathway,” “signal transduction by protein phosphorylation” as well as several terms related to ion transport. However, several of the terms in Table 1 were unexpected including “regulation of inflammatory response,” “negative regulation of cell adhesion,” “negative regulation of cell proliferation,” and “regulation of MAPK cascade.” This finding suggests that tolvaptan has actions that extend beyond the well-known physiological roles of vasopressin.

Table 2 shows GO-BP terms with an over-representation of transcripts that were increased. This table also includes several GO-BP terms that can be viewed as unconventional with regard to the normal physiological effects of vasopressin on collecting duct physiology including “response to transforming growth factor beta,” “positive regulation of epithelial cell proliferation,” “kidney development,” “positive regulation of MAPK cascade,” and “ERK1 and ERK2 cascade.” Both sets of enriched GO-BP terms point to the possibility that tolvaptan may affect MAPK signaling. To investigate this further, we used an antibody to phosphorylated ERK1 and ERK2 to carry out immunoblotting studies. Note that both down- and up-regulated mRNA changes point to the effects of tolvaptan on cell proliferation.

### Regulation of ERK1 and ERK2 Phosphorylation

**Figure 4.**
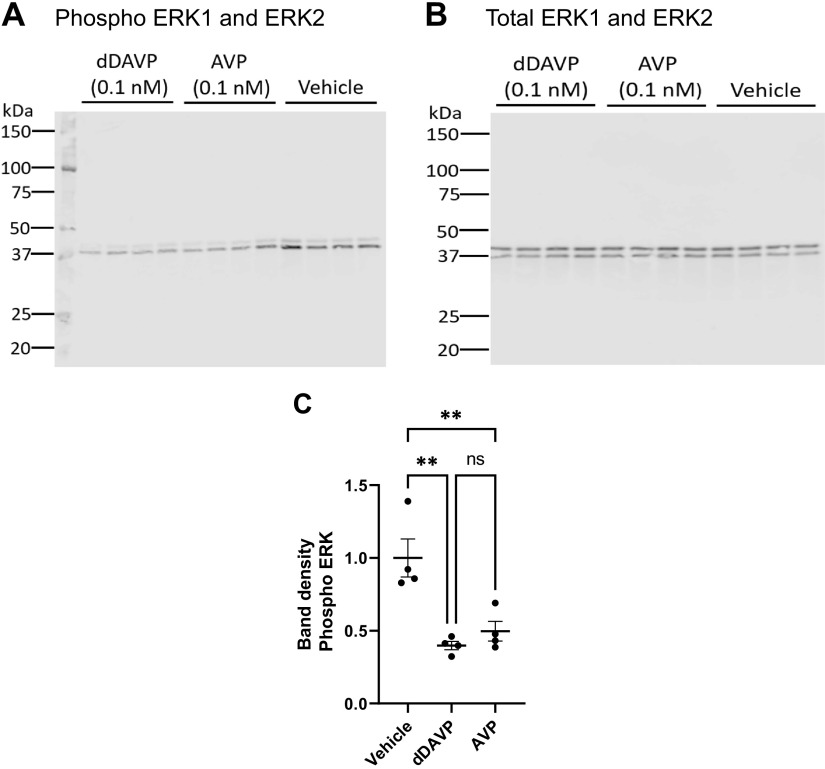
dDAVP and AVP effects on ERK1/2 phosphorylation in mpkCCD cells. *A*: immunoblot with phospho-ERK1/2 antibody. *B*: total ERK1/2. *C*: ordinary one-way ANOVA, overall *P* value <0.0001. **P < 0.001 for individual contrast (Tukey); ns, not significant. AVP, arginine vasopressin.

Cultured mpkCCD cells were grown on permeable supports in the presence of 0.1 nM dDAVP to elicit complete differentiation. A day before the experiment, the dDAVP was washed out. On the day of the experiment, the basolateral medium was replaced by fresh minimal medium with either 0.1 nM dDAVP or 0.1 nM AVP or vehicle and incubated for 30 min (Fig. 4). Figure 4*A* shows the abundances of phospho-ERK1 and phospho-ERK2 for the three conditions (dDAVP, AVP, vehicle), while Fig. 4*B* shows total ERK1 and ERK2. Total ERK1 and ERK2 immunoblots show no significant difference in protein abundance between groups. The phosphorylation of ERK1 and ERK2 was significantly reduced by either AVP or dDAVP compared to the vehicle, but there was no significant difference between dDAVP and AVP responses (Fig. 4*C*). This response is similar to findings in native rat inner medullary collecting ducts showing that dDAVP significantly reduced ERK1 and ERK2 in rat native IMCD cells (10).

**Figure 5.**
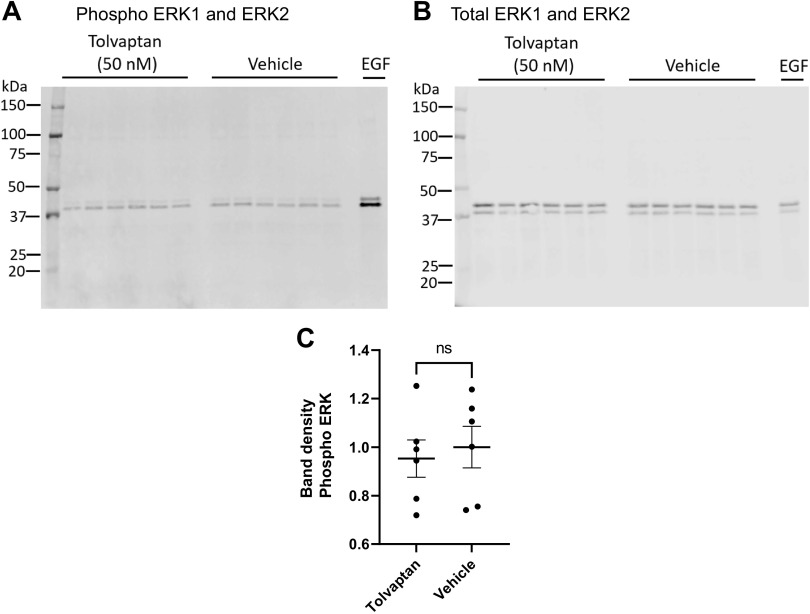
Tolvaptan effect on ERK1/2 activation independently from dDAVP in mpkCCD cells. *A*: immunoblot with phospho-ERK1/2 antibody. *B*: total ERK1/2. *C*: comparison of relative band densities for immunoblot in *A*. Unpaired *t* test showed no significant difference between tolvaptan and vehicle. ns, Not significant.

**Figure 6.**
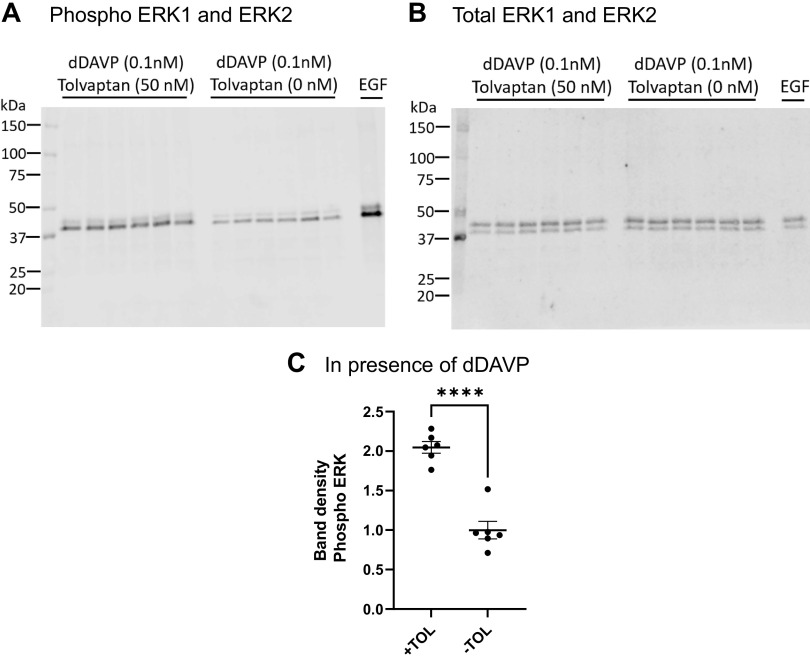
Tolvaptan effect on ERK1/2 activation in the presence of dDAVP in mpkCCD cells. *A*: immunoblot with phospho-ERK1/2 antibody. *B*: total ERK1/2. *C*: comparison of relative band densities for immunoblot in *A*. Unpaired *t* test showed a significant difference between tolvaptan and vehicle in the presence of dDAVP. ****P < 0.0001.

**Figure 7.**
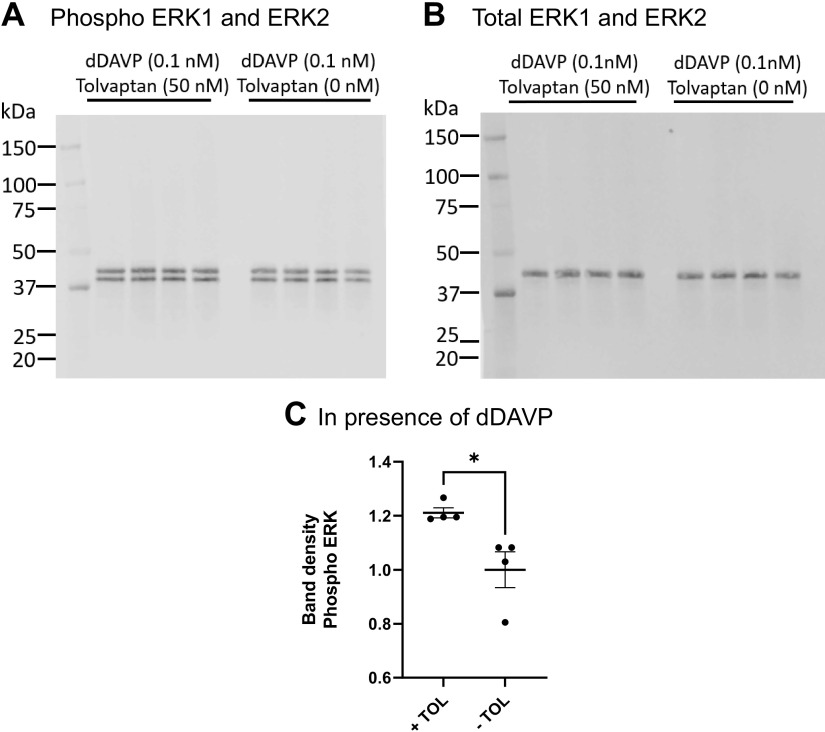
Tolvaptan effect on ERK1/2 activation in the presence of dDAVP in IMCD suspensions from rats. *A*: immunoblot with phospho-ERK1/2 antibody. *B*: total ERK1/2. *C*: comparison of relative band densities for immunoblot in *A*. Unpaired *t* test showed a significant difference between tolvaptan and vehicle in the presence of dDAVP. *P = 0.012, TOL, tolvaptan. IMCD, inner medullary collecting ducts.

Next, we evaluated the effect of tolvaptan in mpkCCD cells in the absence of dDAVP. On the day of the experiment, the basolateral medium was replaced by a fresh minimal medium with no dDAVP and incubated for 30 min in the presence or absence of 50 nM tolvaptan added to the basolateral medium or basolateral 0.1 µM EGF as a positive control (Fig. 5). From this, we conclude that tolvaptan has no substantial effect on ERK phosphorylation in the absence of dDAVP. In contrast, tolvaptan has a strong effect, by increasing ERK1 and ERK2 phosphorylation in the presence of dDAVP (Fig. 6), while not affecting total ERK1 and ERK2. To address this striking finding in native collecting ducts, we further evaluated the effect of tolvaptan in the presence of dDAVP in IMCD suspensions from rats (Fig. 7). Tolvaptan had a significant effect, increasing ERK1 and ERK2 phosphorylation in the presence of dDAVP, while not affecting total ERK1 and ERK2. These findings in native collecting ducts are consistent with the findings in mpkCCD cells.

**Figure 8.**
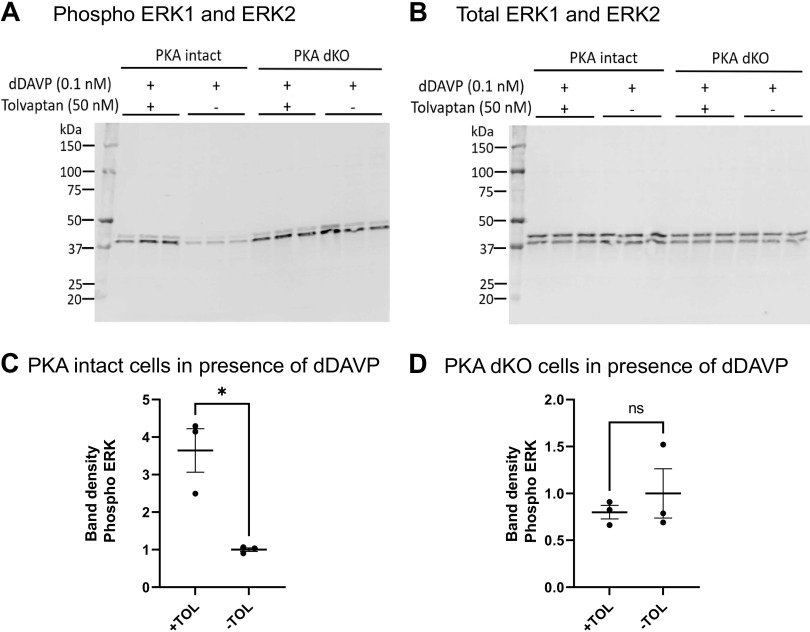
Tolvaptan effect on ERK1/2 phosphorylation in PKA intact and PKA dKO cells in the presence of dDAVP. *A*: immunoblot with phospho-ERK1/2 antibody. *B*: total ERK1/2. *C* and *D*: comparison of relative band densities for immunoblot in *A*. Unpaired *t* test was used to determine the significant difference between tolvaptan and vehicle-treated samples. *P = 0.0103; ns, not significant. PKA dKO, protein kinase A double knockout; TOL, tolvaptan.

**Figure 9.**
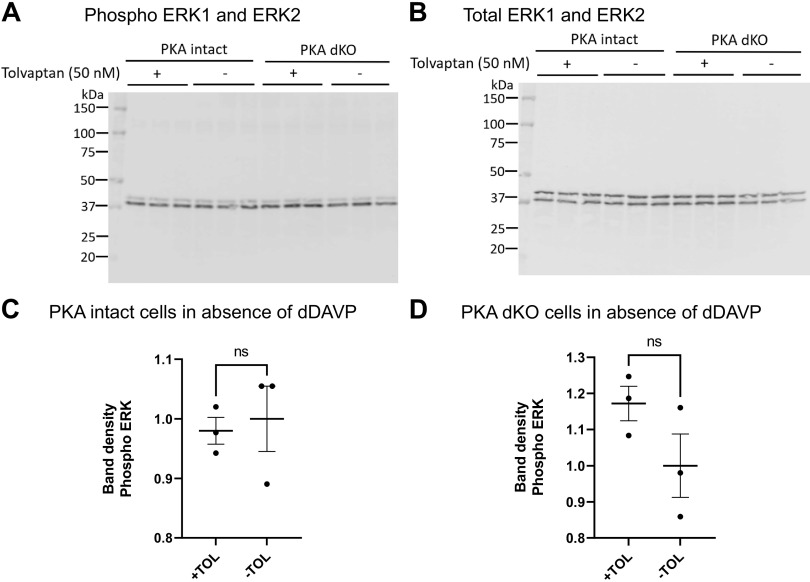
Tolvaptan effect on ERK1/2 phosphorylation in PKA intact and PKA dKO cells. *A:* immunoblot with phospho-ERK1/2 antibody. *B*: total ERK1/2. *C* and *D*: an unpaired *t* test was used to determine the significant difference between tolvaptan and vehicle-treated samples. ns, Not significant; PKA dKO, protein kinase A double knockout; TOL, tolvaptan.

We next addressed whether the effect of tolvaptan to increase ERK1 and ERK2 phosphorylation in mpkCCD cells was due to inhibition of the G_α_s-AC-PKA pathway. To test this, we repeated the experiments in mpkCCD cells in which both PKA catalytic proteins (PKA-Cα and PKA-Cβ) were deleted (PKA dKO) (7). Previous studies (7) with this cell line showed that PKA deletion resulted in a very large increase in ERK1 and ERK2 phosphorylation. As seen in Fig. 8 in the presence of dDAVP, tolvaptan again increased ERK1 and ERK2 phosphorylation in the PKA-intact cells but did not affect ERK1 and ERK2 phosphorylation in the PKA-dKO cells. Thus, it appears that the tolvaptan effect to increase ERK activation is mediated by the G_α_s-AC-PKA pathway. When the same experiment was performed in the absence of dDAVP, tolvaptan again had no significant effect on ERK1 and ERK2 phosphorylation either in the PKA-intact or PKA-dKO cells (Fig. 9).

**Figure 10.**
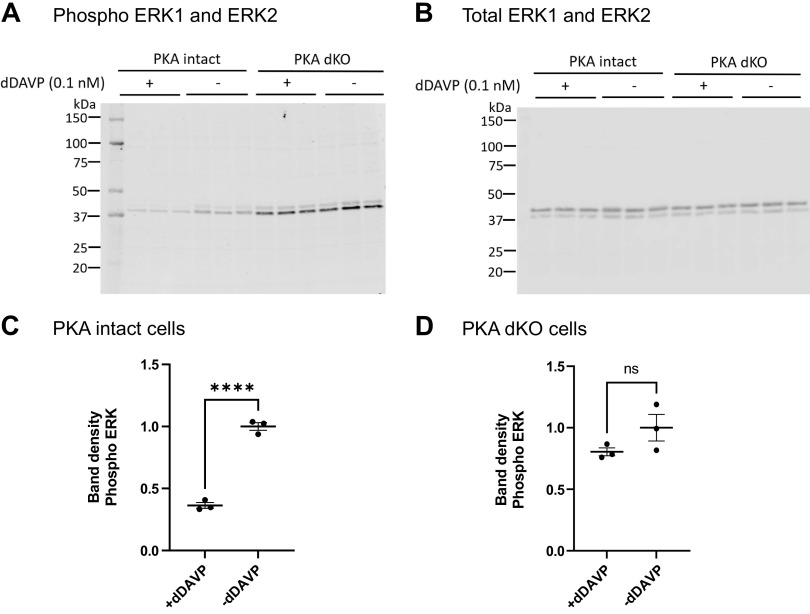
dDAVP effect on ERK1/2 phosphorylation in PKA intact and PKA dKO cells. *A*: immunoblot with phospho-ERK1/2 antibody. *B*: total ERK1/2. *C* and *D*: an unpaired *t* test was used to determine the significant difference between dDAVP and vehicle-treated samples. ****P < 0.001; ns, not significant. PKA dKO, protein kinase A double knockout.

Finally, we evaluated the effect of dDAVP independently of tolvaptan on ERK1 and ERK2 phosphorylation in PKA-intact and PKA-dKO cells, reasoning that if the effect of dDAVP to decrease ERK1 and ERK2 phosphorylation is dependent on PKA, it should disappear in the PKA-dKO cells. The normalized phosphorylation was significantly reduced by dDAVP compared to vehicle in PKA intact cells but not PKA dKO cells (Fig. 10).

One possible explanation for the lack of a PKA-independent effect of tolvaptan to increase ERK1 and ERK2 phosphorylation is that β-arrestin might not be expressed in the mpkCCD cells. To address this possibility, we mined RNA-seq data for the same mpkCCD cell subline used in the present study (48), comparing the values to RNA-seq data from microdissected mouse cortical collecting ducts (40). Both β-arrestin 1 (Arrb1) and β-arrestin 2 (Arrb2) mRNAs were detectable in either the absence or presence of dDAVP, although Arrb2 was more abundant (Table 3). Arrb2 has been shown to be recruited by the vasopressin V2 receptor and promote ERK1 and ERK2 activation (28, 49, 50). Thus, the failure of tolvaptan to increase ERK1 and ERK2 phosphorylation in PKA-dKO cells is not due to the absence of arrestins in collecting duct cells.

**Table 3.** RNA-seq transcripts per million values for mkdCCD cells and microdissected mouse cortical collecting ducts

Gene Symbol	mpkCCD Vehicle 24 h, TPM	mpkCCD dDAVP 24 h, TPM	Native Mouse CCD, TPM
*Arrb1*	0.61	0.45	8.36
*Arrb2*	8.88	8.07	10.05

Data mined from https://esbl.nhlbi.nih.gov/MRECA/Nephron/. TPM, transcripts per million.

## DISCUSSION

To identify the effects of the vasopressin V2 receptor antagonist, tolvaptan, on the transcriptome of collecting duct cells (mpkCCD), we carried out RNA-seq analysis, comparing cells treated with tolvaptan and dDAVP with cells treated with dDAVP alone. The data show that tolvaptan inhibits the expression of mRNAs that were previously shown to be increased in response to vasopressin including AQP2, but also reveals mRNA changes that were not readily predictable from known effects of vasopressin. Some of these effects may be off-target actions of tolvaptan. Bioinformatic analysis of the RNA-seq data pointed to strong effects of tolvaptan to cause changes in the transcriptome associated with activation of the MAPK kinase (ERK1 and ERK2) pathway. Immunoblotting confirmed a strong effect of tolvaptan to increase ERK1 and ERK2 phosphorylation at sites associated with increased kinase activity. A similar increase was seen in suspensions of native rat inner medullary collecting ducts. Tolvaptan-mediated ERK1 and ERK2 phosphorylation increases were not seen in mpkCCD cells in which both PKA catalytic subunits were deleted, indicating that tolvaptan’s effect on ERK activity is PKA-dependent. Based on prior studies in native inner medullary collecting ducts, we speculate that the effect of tolvaptan to stimulate ERK phosphorylation occurs through inhibition of PKA-dependent phosphorylation of Raf1 (10), the upstream component of the RAF-MEK-ERK cascade. In some cell types, the V2 receptor can signal independently of PKA through β-arrestin, which activates ERK1 and ERK2 (13). The lack of effect of tolvaptan on ERK phosphorylation in PKA-null mpkCCD cells suggests, however, that β-arrestin signaling plays little or no role in the regulation of ERK activity in mpkCCD cells.

The vaptan family of V2 receptor antagonists functions as so-called “inverse agonists” (28, 29). An inverse agonist is an antagonist that can block GPCR ligand-induced activation but can also have effects to suppress the constitutive activity of the receptor. Therefore, we speculated that vaptans could have effects independent of vasopressin. However, tolvaptan had no effect on ERK1 or ERK2 phosphorylation in the absence of dDAVP, suggesting that it works chiefly by blocking V2 receptor-ligand binding.

For these studies, we used mpkCCD cells (39), which express the V2 receptor endogenously and have been shown to exhibit vasopressin actions on AQP2 trafficking and transcription that mimic native CCD principal cells (38, 51). The observed vasopressin-induced reduction in ERK1 and ERK2 phosphorylation in mpkCCD cells contrasts with ERK1 and ERK2 phosphorylation increases seen in HEK293 cells overexpressing the V2 receptor (13). Vasopressin-mediated decreases in ERK1 and ERK2 phosphorylation have also been shown previously by mass spectrometry (MS) and immunoblotting of mpkCCD cells (52). We note that in addition to collecting duct principal cells, both thick ascending limb cells and distal convoluted tubule cells also express the V2 receptor (40). We are unaware of any data addressing how vasopressin affects ERK phosphorylation in these cell types. Interestingly, it has been shown that one of the vaptans, namely satavaptan, can act in the absence of vasopressin by recruiting β-arrestin in cells over-expressing the V2 receptor (18, 28). The lack of a vasopressin-independent effect of tolvaptan in mpkCCD cells that expresses the V2 receptor endogenously, appears to rule out such a mechanism in these cells.

One major role of ERK1 and ERK2 is to phosphorylate transcription factors, such as ETS family transcription factors (53–55), AP-1 family transcription factors (56, 57), and E2F family transcription factors (58), leading to changes in gene expression that promote cell proliferation. ERK can also phosphorylate other proteins involved in the cell cycle and mitosis, such as cyclin-dependent kinase inhibitor p27 (59, 60) and topoisomerase 2a (61). Thus, ERK activation can perturb the balance between cell proliferation and differentiation in favor of proliferation.

### Perspectives and Significance

Activation of the V2 receptor by vasopressin is thought to play a critical pathogenic role in autosomal dominant polycystic kidney disease (ADPKD) (62). The loss of function of polycystin-1 or polycystin-2 proteins leads to ADPKD through various signaling pathways (63). V2 receptor signaling results in the stimulation of fluid secretion into growing cysts, in part by stimulation of CFTR-mediated chloride transport, and blockade of V2 receptor signaling by V2R antagonists has the potential of slowing cyst expansion (63). This possibility led to studies testing the use of tolvaptan to slow ADPKD progression, eventually leading to United States Food and Drug Administration (FDA) approval of tolvaptan use for the treatment of patients with ADPKD (64). The current paper was designed to investigate vasopressin action or tolvaptan effects on normal collecting duct cells and not in the setting of ADPKD, but the findings could be relevant to ADPKD. The effect of tolvaptan to markedly increase ERK activity has the potential of having a proliferative effect in ADPKD cells, an issue that should be investigated in future studies.

## DATA AVAILABILITY

RNA-seq data including the raw processing file have been deposited in the Gene Expression Omnibus (GEO, GSE243773, https://www.ncbi.nlm.nih.gov/geo/query/acc.cgi?acc=gse243773). Full-length immunoblots are provided in the main body of this paper.

## GRANTS

This work was primarily funded by the Division of Intramural Research, National Heart, Lung, and Blood Institute (Projects ZIAHL001285 and ZIAHL006129, to M.A.K.). This work was also supported through a National Institutes of Health 2020 Bench-to-Bedside award from the National Institutes of Health Office of Clinical Research (Award No. 654006).

## DISCLOSURES

No conflicts of interest, financial or otherwise, are declared by the authors.

## AUTHOR CONTRIBUTIONS

S.K., V.R., and M.A.K. conceived and designed research; S.K. performed experiments; S.K. and M.A.K. analyzed data; S.K., V.R., C.-L.C., C.-R.Y., S.J.K., and M.A.K. interpreted results of experiments; S.K. prepared figures; S.K. and M.A.K. drafted manuscript; S.K., V.R., L.C., C.-L.C., C.-R.Y., S.J.K., and M.A.K. edited and revised manuscript; V.R., L.C., C.-L.C., C.-R.Y., S.J.K., S.K., and M.A.K. approved final version of manuscript.
